# Roles of zinc and zinc transporters in development, progression, and treatment of inflammatory bowel disease (IBD)

**DOI:** 10.3389/fnut.2025.1649658

**Published:** 2025-09-24

**Authors:** S. B. Mitchell, T. B. Aydemir

**Affiliations:** Division of Nutritional Sciences, Cornell University, Ithaca, NY, United States

**Keywords:** ZIP14, ZIP8, Crohn’s disease, ulcerative colitis, zinc deficiency (ZnD), zinc supplementation (ZnS), intestine, colon

## Abstract

The inflammatory bowel diseases (IBD), including Crohn’s Disease and Ulcerative Colitis, are chronic, incurable disorders of the gastrointestinal tract. These multifactorial diseases pose an enormous burden on patients, clinicians, and public health systems worldwide. Zinc (Zn) is an essential micronutrient that is required for a wide variety of functions critical to maintaining gastrointestinal health. Zn homeostasis is facilitated by the SLC39/ZIP and SLC30/ZnT families of solute carrier proteins, which collectively distribute Zn with subcellular specificity. Disruptions in Zn homeostasis can have substantial impacts on health, as recent years have seen Zn transporters become increasingly recognized for their importance in health and disease. Although dietary Zn deficiency is rare in the United States, Zn deficiency is common among IBD patients. Disruptions in Zn homeostasis have also been shown to play a role in the progression of IBD. Despite these links, Zn supplementation trials in IBD have shown inconsistent results. This review focuses on the role of Zn and Zn transporters in the development, progression, and treatment of IBD, as well as discussing the challenges and potentially promising future of the study of Zn and Zn transporters in precision health.

## 1 Introduction

Zinc (Zn) is an essential micronutrient required by all forms of life for survival. The cellular and molecular roles of Zn are diverse, including structural roles in proteins, acting as a cofactor in enzymes, catalytic activity, regulation of gene expression, and participation in cell signaling pathways. Zn, the gastrointestinal tract, and human health are deeply interconnected. As the primary site of absorption of dietary Zn, the small intestine provides Zn for all cells in the body. In turn, Zn is required for processes essential to maintaining the health of the gastrointestinal tissues, including the maintenance of epithelial barrier integrity, cellular growth and proliferation, and the production of the mucus layer.

Systemic and local Zn homeostasis is controlled by the SLC39A/ZIP and SLC30A/ZnT families of transporters. Together, these transporters facilitate Zn trafficking with tissue-, cellular-, and subcellular-specificity. Abnormalities or disruptions in Zn homeostasis can, therefore, pose great risks to human health. Indeed, recent years have seen Zn transporters become recognized for their importance in health and disease, having been implicated in chronic diseases, including inflammatory bowel disease (IBD). As a multifactorial, incurable disease, the exact causes of IBD remain unknown and vary between individuals but are thought to include an individual’s genetic susceptibility, environmental factors such as diet, gut microbiome, and host immune responses. Despite the heterogeneous nature of IBD, Zn is known to be involved in each of these hallmarks of disease. Zn is involved in the regulation of host gene expression, and individual expression of genes related to Zn homeostasis, including Zn transporters, has been implicated in IBD. Furthermore, zinc is essential for modulating immune responses and regulating gut microbial composition.

This review examines the role of Zn and Zn transporters in the context of IBD. First, we discuss Zn, Zn deficiency, and Zn transporters in the context of health and disease. We then explore the interplay between Zn and IBD, including epidemiological studies, clinical interventions, and studies in cellular and animal models. Finally, we discuss remaining challenges and emerging opportunities for further research. Collectively, we aim to provide a current understanding of the role of Zn and Zn transport in IBD, highlighting the importance of these studies as we aspire toward potential applications in precision nutrition and medicine to meet modern health needs.

### 1.1 Zinc

Zn is an essential micronutrient and trace element required for all forms of life. Zn is required for a wide variety of cell processes, including structural and catalytic roles in proteins, gene regulation, and signaling processes. As a catalytic cofactor, zinc is involved in all six enzyme classes (oxidoreductases, transferases, hydrolases, lyases, isomerases, and ligases), either directly driving bond-making and bond-breaking reactions at the active site (catalytic zinc) or enhancing enzymatic activity in multi-metal systems, where zinc collaborates with other metal ions positioned in close proximity, one serving a catalytic role while others modulate activity (cocatalytic zinc) (1–5). Structurally, zinc stabilizes the tertiary and quaternary architectures of proteins, including zinc-finger domains in transcription factors, and facilitates protein–protein, protein-DNA/RNA, and protein-lipid interactions (6–9). Zinc also modulates gene expression by binding to metal-response element-binding transcription factor-1 (MTF-1), which activates or represses specific target genes in response to zinc availability (10–12). Additionally, zinc functions as a signaling molecule, acting as a first or second messenger to regulate diverse signaling pathways, including NFκB activation, apoptosis, and ion-channel activity (13–16). These multifaceted roles enable zinc to integrate enzymatic function, structural integrity, and signaling dynamics across numerous physiological processes.

Despite the essentiality of Zn and its diverse roles in cells and tissues, Zn was not recognized as important for human nutrition until relatively recently. As late as the 1960s, there was a belief that Zn deficiency could not exist in humans due to the fact that Zn is nearly universally present in human foods. This began to change in 1961 with the observation that the administration of a hospital diet reversed stunted growth and hypogonadism in a series of case studies of Iranian teenage boys and young men with a history of geophagia and poor-quality diets (17). Today, it is recognized that approximately 17% of the world’s population, over 1 billion people, are at risk of inadequate Zn intake (18). Despite now more than 60 years of research focusing on Zn in human health and nutrition, many mechanisms of action of Zn remain unclear due to the widespread and interconnected roles of Zn in cells and tissues. This makes the study of Zn and its transport in modern health and disease both greatly important and exciting.

Zn is the second most abundant trace element in humans, behind iron (Fe). In total, there is between 1.5 – 2.5 g of Zn in an average person, with approximately 57% in residing in skeletal muscle, 29% in bone, 6% in skin, 5% in liver, and only 0.1% in blood plasma (19, 20). These, of course, are estimations; however, the disparity in Zn content between different tissues is useful for highlighting several important aspects of Zn physiology. First, the low proportion of body Zn found in circulation has implications for assessing body Zn status, which is primarily determined by measuring plasma or serum Zn. This means that measurements of Zn status may not reflect Zn status in tissues of interest. Second, these disparities indicate that small changes in tissue Zn content in muscle or liver tissue can have a significant impact on circulating Zn status. Nearly all body Zn is intracellular, with about 50% of that in the cytoplasm, 40% in the nucleus, and 10% in cellular and organelle membranes (20). Total cellular Zn concentrations vary but are thought to be approximately 250 μM (21, 22). The specific and disparate distribution of Zn at the tissue and intracellular level is itself evidence that Zn homeostasis in humans is tightly controlled. Furthermore, it highlights how changes in Zn distribution, induced by diet or disease, can have significant impacts on health.

Zn deficiency can be dietary or acquired. Zn deficiency can result in growth stunting, affecting the skin, gastrointestinal tissues, the immune system, the skeletal system, and the reproductive systems most severely (20). For adults, the Recommended Dietary Allowance is 11 mg/day for men and 8 mg/day for women (20, 23). Dietary Zn deficiency is most common in countries in South and Southeast Asia, Sub-Saharan Africa, and Central America, based on Zn availability in their national food supplies (18). Symptoms include stunted growth, wasting, impaired immunity, and impaired sexual maturation (18, 20, 23). Outright dietary Zn deficiency is rare in the United States, with vegetarians and women who are pregnant or lactating at the greatest risk for dietary inadequacy due to bioavailability and increased requirements, respectively (24, 25). Zn bioavailability can be influenced by presence of Zn chelators in foods. This most notably occurs via phytic acid, or phytates, found in seeds, grains, cereals, and legumes, which is known to bind dietary Zn, rendering it unavailable for dietary absorption. In fact, the phytate/zinc molar ratio is used by global health organizations to assess the quality of Zn bioavailability in foods (26, 27). Conversely, other naturally occurring Zn chelators such as carnosine can improve Zn availability and may be useful for treatment of gastrointestinal disorders such as *H. pylori* infection and associated ulceration (28).

Acquired Zn deficiency includes deficiency resulting from disease, most notably acrodermatitis enteropathica, a fatal skin disease caused by a mutation in ZIP4 resulting in Zn malabsorption (29, 30). Additionally, Zn deficiency can be induced by inflammatory diseases such as diabetes and infections. Diabetes in particular is a powerful example of modern diseases altering Zn homeostasis, resulting in deficiency. In patients with diabetes, urinary excretion of Zn is increased, resulting in a circulating Zn deficiency (31, 32). This is of great concern, as Zn is essential for insulin production and secretion (8, 33). Indeed, oral supplementation with Zn has beneficial effects on glycemic control and lipid parameters in patients with diabetes (34). This illustrates how disease-induced alterations in Zn homeostasis can, in turn, negatively impact disease outcomes, forming a self-perpetuating cycle of disease-Zn interactions. As chronic inflammatory and metabolic disorders are among the defining health challenges of our time, clearly defining the interactions between disease and Zn homeostasis is essential for optimized treatment and management of these diseases.

Excessive amounts of Zn can be toxic to cells in *in vitro* settings; however, in practice, dietary Zn toxicity is not a significant concern in humans. Cases of toxicity can occur following prolonged high-dose supplementation or occupational exposure, such as in industrial settings, which primarily manifests as a copper deficiency (20, 35). In rare cases, allergy to Zn has been observed in individuals, particularly in association with insulin. In some individuals who experienced an allergic response to insulin injections, Zn-free insulin did not induce an allergic response (36, 37).

Assessment of Zn in humans continues to be a challenge. Zn deficiency can go undetected as there is no specific biomarker for Zn that reliably and sensitively indicates an individual’s Zn status (38). The most commonly used indicators for Zn status are plasma or serum Zn concentrations (PZC) (39). These assessments are useful for detecting moderate to severe dietary deficiency and responses to supplementation. PZC is also associated with clinical signs of deficiency, such as growth (39). However, there are major limitations to PSCs as indicators of Zn status, especially when trying to evaluate Zn status in chronic disease. In the presence of inflammation, PZCs are reduced, potentially resulting in an incorrect assessment of Zn deficiency (40, 41). To this end, efforts such as those by the Biomarkers Reflecting Inflammation and Nutritional Determinants of Anemia (BRINDA) project have been made to adjust or correct PZCs for inflammation based on correlations with C-reactive protein or α-1-acid glycoprotein. However, these adjustments were not found to be necessary (41). Circulating Zn levels also fluctuate around recent intake due to lack of storage. PZCs can be further impacted by a variety of factors, including practical considerations such as the hemolysis of collected samples, as well as factors like the time of day of sample collection and the timing since the last meal. Additionally, circulating Zn concentrations may not accurately reflect local tissue Zn status in areas of interest due to the small proportion of body Zn in circulation. This presents a challenge in accurately determining which individuals are most likely to benefit from Zn-based therapies. Recent efforts to improve the assessment of Zn status have taken a multiparameter approach. The Biomarkers of Nutrition for Development (BOND) project recommends estimating the Zn status of populations by considering three indicators: the prevalence of Zn intakes below the estimated average requirement, the percentage of the population with low PZCs, and the percentage of children aged years and younger who are stunted (39). For the assessment of individuals, a three-parameter formula has been proposed as a Zn status index (ZSI). The formula consists of the mRNA gene expression of Zn-related proteins, fecal microbiome profiling, and the linoleic acid:dihomo-*γ*-linolenic acid (LA: DGLA) ratio, for which Zn is essential for the enzymatic conversion of LA to DGLA by Δ6 desaturase (42). These assessments could then be used to estimate the probability of Zn adequacy, allowing for the classification of Zn status ranging from severely deficient to adequate. Ultimately, there remains no ‘gold-standard’ marker for Zn status and more research is required to clearly define the biological roles of Zn, particularly in the context of disease. Improved understanding of the interactions between Zn homeostasis and chronic disease as well as more accurate measurement of Zn status in individuals is essential for precision health and nutrition approaches.

### 1.2 Zn transport

Zn status is tightly regulated by two families of transport proteins, the ZnT/SLC30 and ZIP/SLC39 families of solute carrier proteins. The ZIP/SLC39 family comprises 14 members (ZIP1-14) and functions to transport Zn into the cytosol of cells, either by uptake from outside the cell or by releasing Zn from organelles. Conversely, the ZnT/SLC30 family comprises 10 members (ZnT1-10) that transport Zn out of the cytosol and into organelles or export Zn out of the cell (43, 44). Together, these 24 transporters traffic Zn to cellular and subcellular specificity, facilitating the targeted functions of Zn. These transporters are diverse in their localization, function, and regulation. These transporters only began to be characterized in mammals near exactly 30 years ago. In 1995, Palmiter and Findley characterized ZnT-1 by cloning rat cDNAs that conferred Zn resistance to baby hamster kidney cells (45). The proceeding 30 years to date have seen the study of specific Zn transporters in human disease become of great interest. Two examples are ZIP4 and ZnT8. In humans, ZnT8 is primarily found in pancreatic beta cells, where it transports zinc required for the incorporation into insulin granules (8, 46–48). Indeed, ZnT8 has been identified in genome-wide association studies as a risk locus for type 2 diabetes (49, 50). Perhaps the classic example of Zn transporters in human disease is the aforementioned acrodermatitis enteropathica, a fatal skin disease that predates knowledge of Zn in human nutrition (29). It has since been found to be caused by a mutation in ZIP4, which is localized to the apical membrane of enterocytes and is primarily responsible for the absorption of dietary Zn (30). This provides an extreme but useful example of the essentiality not just of Zn, but of the specific transport proteins that facilitate its movement to target tissues and cells.

**Figure 1:**
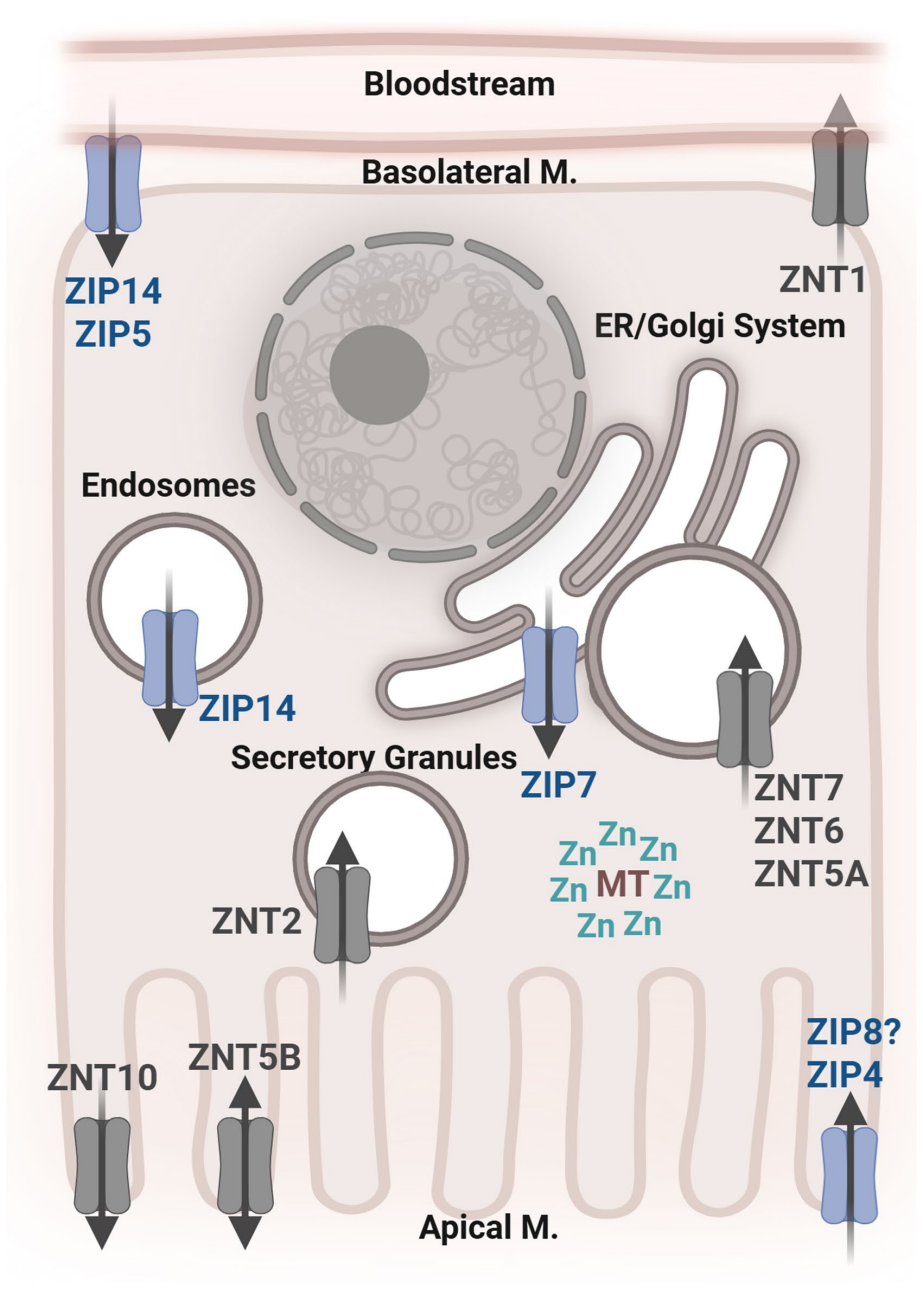
General depiction of an intestinal epithelial cell with the localization of known Zn transporters. ZIP, Zrt-Irt-Like Proteins; ZNT, Zinc Transporter; MT, Metallothionein; Zn, Zinc; M, Membrane; ER, Endoplasmic Reticulum.

Zn transport and homeostasis begin with the absorption of dietary Zn by the small intestine (Figure 1). Though it is absorbed throughout the entire small intestine, the jejunum displays the highest rate of Zn absorption (44, 51). ZIP4 is expressed abundantly throughout the small intestine and is considered the primary transporter responsible for the absorption of dietary Zn (44). Following absorption into enterocytes, the transporter ZnT1 moves Zn into circulation (20, 52). This movement of Zn from the lumen into circulation has been the subject of most research on Zn transport. However, transport of Zn in the opposite direction does occur. Zn is excreted primarily through the feces. That is, endogenous Zn is transported from circulation back across the gut epithelium and into the lumen (20, 53). Secretion of Zn from the pancreas and transepithelial Zn transport are the two main routes of movement of endogenous Zn into the lumen. Transepithelial Zn transport in the serosal-to-mucosal direction is thought to be primarily mediated by ZnT5, ZnT10, ZIP5, and ZIP14. On the basolateral membrane, ZIP5 and ZIP14 transport Zn from the circulation into epithelial cells (44, 54). ZnT5 and ZnT10 release Zn from epithelial cells into the gut lumen (55–57). ZnT5B, a variant of ZnT5, has been shown to mediate bidirectional Zn transport in human intestinal Caco-2 cells (57). The transepithelial route of Zn transport is becoming increasingly recognized as critical for maintaining systemic and gastrointestinal Zn homeostasis. Therefore, the study of Zn transporters that are localized to the basolateral membrane of intestinal epithelial cells and their associated transport of Zn is of great interest, particularly in the context of disease.

One such transporter is SLC39A14/ZIP14, which is one of the most well-characterized ZIP transporters, with known roles in the liver, adipose tissue brain, bone, and intestinal tissues (54). The roles of ZIP14, specifically in the intestine, will be discussed later on. In addition to Zn, ZIP14 can transport nontransferrin-bound Fe, manganese (Mn), and cadmium (58, 59). Though at physiological concentrations, the preferred substrate of ZIP14 transport is Zn (60). It is thought to be localized on the plasma membrane of cells (59, 61–63). ZIP14 is expressed in many mammalian tissues throughout the body, with the greatest abundance in the small intestine, liver, heart, and pancreas (16, 59, 62). It is not thought to be regulated by Zn levels or dietary Zn intake but rather by proinflammatory cytokines. In the liver, ZIP14 was found to be induced specifically by IL-6 and IL-1β (54, 62, 64) This sensitivity to inflammatory conditions is a notable feature of ZIP14 among other Zn transporters, making it a prime candidate for the study of Zn homeostasis in modern chronic inflammatory diseases (54, 65).

### 1.3 Inflammatory bowel disease

The inflammatory bowel diseases are ulcerative colitis and Crohn’s disease, which are chronic, uncurable inflammatory diseases involving the gastrointestinal tissues. These diseases differ in their localization and presentation, with Crohn’s disease occurring in discontinuous or patchy patterns anywhere from the oral cavity to the anus, affectionately referred to as the “gum to bum” pattern. Ulcerative colitis is continuous in its presentation and is localized to the large bowel or colon. Crohn’s disease causes transmural inflammation, while ulcerative colitis is restricted to the mucosa and submucosa (66).

Patients suffering from IBD experience repeating cycles of inflammation of the gastrointestinal tract. Rectal bleeding occurs in more than 90% of patients with ulcerative colitis (67). Abdominal pain, bloody feces, and diarrhea, both increases and decreases in bowel movement frequency, fecal incontinence and mucus discharges are among the most common symptoms of ulcerative colitis. Both ulcerative colitis and Crohn’s disease can present with extraintestinal manifestations, which may even precede symptoms in the gastrointestinal tissues. The most common of these include peripheral arthritis, skin diseases, and renal and pulmonary manifestations (67, 68).

The specific causes of IBD are unclear and likely to vary greatly between individuals. However, it has become clear in recent years that a key component of disease development is a loss of tolerance to commensal microbiota, resulting in a dysregulated immune response (66, 69). This includes many factors, such as genetic susceptibility, environmental and dietary considerations, and host–microbe interactions (69–72). A loss of barrier integrity is a hallmark of IBD. The disruption of the intestinal barrier, resulting in increased gut permeability, is a predictor of disease onset and relapse (73–75). The link between gut permeability and IBD extends beyond the individual, as even first-degree relatives of Crohn’s disease patients have been found to have increased intestinal permeability (76).

The diverse presentations and experiences of IBD from individual to individual have made treatment and management of the disease difficult for both patients and clinicians. Upon diagnosis, patients are sorted into Crohn’s disease and ulcerative colitis based on clinical observations described previously. This results in groups of patients with likely very different drivers of the disease being classified in the same way. Accordingly, there has been an effort to identify personalized care for IBD patients (77). Genome-wide association studies have identified hundreds of risk loci associated with IBD, but the mechanistic contributions of these loci remain largely unknown (77, 78). In addition to host genetics, changes in the microbiome, both in its composition and functional capacity, have been linked to IBD. Although the body of knowledge regarding the likely mechanisms of disease development has greatly improved in recent years, there has been limited success in new therapeutic development or measurable differences in prognosis. Ultimately, the numerous factors that contribute to IBD and the immense individual variability in disease onset and presentation necessitate research that accounts for both host genetics and microbial contributions, as well as the interactions between them, to develop more effective therapeutic approaches.

As a low-mortality and incurable disease, IBD continues to place an increasing burden on patients and healthcare systems globally. IBD has traditionally been more prevalent in Western and developed countries but is now increasing more rapidly in developing countries. Globally, there are estimated to be between 5 and 7 million cases of IBD (79, 80). Given that the most common age of onset for IBD is in the second and third decades of life, patients often experience IBD for the majority of their life. Collectively, this places an enormous burden of treatment and management on patients, healthcare providers, and healthcare systems.

Arguably, the most important point of contact between the human host and the external environment is the gut epithelium. This barrier is responsible for both the absorption of dietary nutrients and the prevention of the passage of microbes and antigens. Achieving this requires a well-coordinated symphony of tight junctions, the secretion of mucus and antimicrobial peptides, and immune regulation. When inflammatory responses at the intestinal barrier become dysregulated, disease can occur. An underactive or defective inflammatory response can lead to infections caused by harmful pathogens, while an overactive or uncontrolled inflammatory response can lead to diseases such as IBD. Disruption of the barrier allows pathogens and other antigens to cross into the bowel wall, leading to an immune response. During and between periods of inflammation in IBD, the intestinal barrier must undergo a healing process in order to restore normal function and achieve remission. Healing, or regeneration, of the barrier involves re-establishing the integrity of the barrier so that antigens can no longer cross (81, 82). Indeed, mucosal healing has been a critical goal of IBD therapeutics (83). Achieving this goal is associated with improved prognosis in both ulcerative colitis and Crohn’s disease (84).

There have been numerous dietary factors and specific foods that have been associated with increased progression or incidence of IBD. These include meat intake, sugar intake, consumption of ultra-processed foods, dietary fat intake, and Western dietary patterns (85–89). Likewise, many dietary factors have been linked to a reduced risk of disease development, including Mediterranean diets, fruit and vegetable intake, and dietary fiber intake (90–92).

As a chronic disease impacting the gastrointestinal tract, it is not surprising that nutrient deficiencies commonly occur alongside IBD. In some of the earliest descriptions of illnesses that would eventually become known as IBDs, emaciation, and wasting were significant clinical observations (93). In fact, the original description of “regional ileitis” by Crohn, Ginzburg, and Oppenheimer in 1932 states, “The disease is clinically featured by symptoms that resemble those of ulcerative colitis, namely, fever, diarrhea, and emaciation” (94) It is now understood that factors, including reduced food intake, malabsorption, and increased nutrient requirements, contribute to the development of malnutrition and nutrient deficiencies in IBD (95, 96) Up to 85% of patients experience some form of malnutrition, with both macronutrient and micronutrient being common. The most common micronutrient deficiencies include Fe, vitamin B12, vitamin D, and Zn (95, 96).

### 1.4 Zn and IBD

Associations between Zn and IBD are plentiful. Although dietary Zn deficiency is rare in the United States, up to 50% of IBD patients may be Zn deficient (95, 97, 98). Further, Zn status and dietary Zn intake are inversely associated with both the risk for disease development and the severity of symptoms and complications (99–101). Zn deficiency has been associated with an increased risk of hospitalizations, surgeries, and other complications (101). A deficiency of Zn in IBD could arise from multiple factors, most notably dietary deficiency, which may result from dietary insufficiency, malabsorption due to other dietary factors (e.g., phytates), or genetic malabsorption. Additionally, deficiency could arise from disease-induced (non-dietary) conditions such as damaged absorptive tissues. However, it remains unclear whether Zn deficiency is a symptom or driver of disease development.

Aside from epidemiological studies showing associations between Zn intake and status and IBD, there have been attempts to utilize Zn supplementation in the treatment and management of IBD. These have yielded mixed results, with some studies showing improvements in disease activity while others have shown no significant results (Table 1). In a small cohort of Crohn’s disease patients who had been in remission for at least 3 months, high-dose oral Zn sulfate supplementation for 8 weeks reduced intestinal permeability (102). In a different study, IBD patients with Zn deficiency were given oral supplementation of 25-150 mg/day Zn acetate hydrate. In patients with Crohn’s disease, both Zn deficiency and the Crohn’s disease activity index improved after 4 and 20 weeks. However, patients with ulcerative colitis did not consistently improve their Zn levels or disease activity, as measured by the partial Mayo score, after 4 or 20 weeks of supplementation (103). Patients with ulcerative colitis who received nutritional guidance promoting a Zinc-Rich diet showed improvements in disease activity scores after 24 weeks compared to those who did not receive guidance (104). Administration of Zn carnosine by enema showed significant improvement in endoscopic scores and induction of remission (105). Patients with ulcerative colitis and Zn deficiency were supplemented with 30 mg/day of Zn gluconate for 60 days. Plasma Zn levels increased, but the intervention failed to induce activity of superoxide dismutase in red blood cells (106). Ulcerative colitis patients given 35 mg/day Zn gluconate for 30 days displayed increased circulating Zn but did not show changes in circulating concentrations of TNF-*α*, IL-6, or IL-10 (107). A similar study where ulcerative colitis patients with low circulating Zn received 35 mg/day Zn gluconate for 60 days showed increases in circulating Zn and reductions in IL-2 and IL-10, but no significant changes in IL-4, IL-6, IL-17, TNF-α, or IFN-*γ* (108). Ultimately, Zn interventions in IBD can effectively increase circulating Zn levels; however, improvements in markers of inflammation, histopathology scores, or disease activity scores do not consistently accompany this increase. These discrepancies may be due to differences in treatment dose and duration, route of administration, different forms of Zn provided, current disease state of the participants, and differing individual factors between patients. This also highlights that assessment of circulating Zn levels may not be reflective of disease state or severity in IBD.

**Table 1:** Summary of the clinical trials to test the effect of Zn Supplementation on IBD.

Disease type and stage	Dose and form of Zn	Duration	Control (n)	Treatment (n)	Year	Outcome	Citation
UC-Active disease	Zn Sulfate-220 mg/3Xday	4 weeks	25 (glucose capsules)	26	1977	No benefit as an adjuvant treatment.	(150)
IBD-Mixed stages	Zn Aspartate-100 mg/3Xday	8 weeks (at 4 weeks crossed over)	6	7	1993	Trends toward reducing natural killer cell activity.	(151)
CD-Clinical remission	Zn sulfate-200 mg/day	6 weeks	20	20	1994	Improved Zn status and abnormal erythrocyte membrane long-chain fatty acid composition.	(152)
IBD-Active disease	Zn Aspartate-300 mg/day	4 weeks	22	14	1994	Zn as an adjuvant treatment improved Zn status but had no effect on plasma, erythrocyte, or mucosal metalloprotein levels.	(153)
CD-Clinical remission	Zn Sulfate-110 mg/3Xday	8 weeks		12 (pre & post)	2001	Reduced intestinal permeability.	(102)
UC-Clinical remission	Zn Gluconate -30 mg/day	3 months	12	12	2014	Improved Zn status, but no change in superoxide dismutase activity.	(106)
UC-Mixed stages	Zn gluconate -35 mg/day	1 month	18	23	2018	Improved Zn status, but no significant change in serum inflammatory markers.	(107)
UC-Mixed stages	Zn gluconate-35 mg/day	2 months	18 (corn starch capsules)	23	2020	Improved Zn status and attenuating inflammatory markers, IL2 and IL10.	(108)
IBD-Zn deficiency in mixed stages	Zn acetate -25-150 mg/day	4 and 20 weeks		40CD & 11UC (pre & post)	2022	Zn as an adjuvant treatment improved Zn status and the CDAI scores in patients with CD. Zn as an adjuvant treatment improved Zn status, but no change in Mayo scores in patients with UC.	(103)
UC-Mildly active disease	A diet rich in Zn and n-3 fatty acids	12-24 weeks	10	10	2023	Adjuvant dietary intervention rich in zinc and n-3 fatty acids improved disease severity, resulting in significantly higher clinical remission rates.	(104)

IBD, Inflammatory Bowel Disease; UC, Ulcerative Colitis; CD, Crohn’s Disease; Zn, Zinc; CDAI, Crohn’s Disease Activity Index.

Of the IBDs, Zn deficiency has been most well-established in Crohn’s disease, having been documented since at least 1980. This is thought to be primarily caused by reduced absorption of dietary Zn, even when the tissue appears normal or even in remission (109–112). Comparatively, Zn deficiency is less common in ulcerative colitis. As ulcerative colitis does not present in the small intestine, the primary site of Zn absorption, the complications with Zn absorption often found in Crohn’s disease are typically not a concern in ulcerative colitis. If systemic Zn deficiency in ulcerative colitis does arise, it is likely driven by decreased intake of Zn due to the active illness. In these cases, there are measurable negative impacts of Zn deficiency. However, measurements of systemic Zn (i.e., circulating Zn levels) may not accurately reflect tissue Zn status in patients with ulcerative colitis. The concept of local Zn deficiency in ulcerative colitis is supported by findings of lower metallothionein expression in patients with ulcerative colitis compared to healthy control patients (113). However, this area remains nearly entirely unexplored, and measurements of systemic or circulating Zn remain the most common. Furthermore, the contributions of the colon to Zn homeostasis are largely unknown. Zn absorption occurs primarily in the small intestine, but when absorption is impaired, such as in CD, whether colon absorption or excretion of Zn may compensate is unknown.

Some work has been done to explore how host Zn status may influence IBD development in mouse models of colitis. High-dose Zn supplementation in mice for 2 weeks prior to the induction of colitis by either dextran sodium sulfate (DSS) or 2,4,6-trinitrobenzene sulphonic acid (TNBS) resulted in reduced disease severity (114). In TNBS colitis, Zn deficiency induced by a cell-permeable Zn chelator N, N, N′, N′-tetrakis(2-pyridylmethyl)ethylenediamine (TPEN) was found to aggravate colonic inflammation by activating type 17 helper T (Th17) through the activation of IL-23 (115). Conversely, high dose supplementation of Zn through the diet protected mice from TNBS-induced colitis, at least in part by reducing Th17 cells (116). In rats, dietary Zn deficiency has been found to exacerbate DSS-induced colitis (117, 118). Treatment with Zn oxide nanoparticles alleviated colitis symptoms in DSS colitis in mice by suppressing reactive oxygen species production through activation of Nrf2 (119). Zn supplementation by oral gavage of Zn sulfate in rats improved body weight and reduced the frequency of diarrhea but did not improve colonic inflammation or damage (120, 121).

Mechanistically, Zn is known to be critical for processes essential to maintaining the health of the gut barrier. As the small intestine is the primary site of absorption of dietary Zn, there is an interconnectivity between local and systemic Zn homeostasis and gastrointestinal health (122). Zn is known to regulate gut barrier integrity by altering the expression of tight junction proteins. In Caco2 cells, a pharmacological Zn deficiency induced by TPEN resulted in increased permeability by downregulating claudin-2 and occludin (123). Supplementation of Zn in Caco2 cells has also been found to improve cell growth and barrier integrity through the activation of the PI3K/AKT/mTOR pathway (124). In a human goblet cell model, HT-29-MTX cells made Zn deficient displayed impaired mucin production, mostly from changes in post-translational modifications. Zn-deficient cells were found to have fewer and shorter O-glycans (125). In piglets, Zn supplementation increased the expression of tight junction proteins, including zonula occludens-1 (ZO-1) and occludin, as well as the enzyme total superoxide dismutase (T-SOD), for which Zn functions as a cofactor (126). Meanwhile, the expressions of interleukin-1β and IFN-*γ* were decreased by Zn supplementation (127).

Zn may also participate in nutrient-nutrient interactions that influence gastrointestinal health. In a DSS model of colitis, dietary Zn was required for the activation of the aryl hydrocarbon receptor (AHR) to have a beneficial effect on disease outcomes. Without sufficient Zn, activation of AHR by its dietary ligand indole-3-carbinol (I3C) failed to improve barrier integrity and overall disease outcomes. The combined treatment of both Zn and I3C showed the greatest improvements in barrier integrity, mucin expression, and histological scoring (128). In a different study, a combined treatment of Zn, prebiotics, and probiotics improved disease outcomes in DSS-induced colitis by a greater degree than the probiotic intervention alone. The combined treatment reduced the expression of TNFα, IL-6, IL-1β, and IL-17 while increasing the expression of IL-10, suggesting that the combined therapeutic effect was mediated through the modulation of inflammatory responses (125). These findings suggest that Zn is not only important for its independent functions related to the health of gastrointestinal tissues but also that it may interact with other dietary or environmental factors to promote health. Further study into the relationships between Zn and other dietary factors may therefore be a fruitful route for improving our understanding of how dietary factors influence gastrointestinal health and the risk of chronic disease.

### 1.5 Zn transporters and IBD

The study of Zn transporters in IBD is still in its early stages, though some studies have begun to identify potential roles. Most notably, a missense variant of SLC39A8/ZIP8, rs13107325, has been associated with an increased risk of Crohn’s disease. This single-nucleotide polymorphism, which encodes either alanine or threonine at position 391, is also associated with altered microbiome composition, indicating a potential microbiome-driven role for ZIP8 in Crohn’s disease (129). A mouse model of this ZIP8 variant (SLC39A8 A393T) demonstrated that the altered ZIP8 resulted in increased cobalt in the colonic mucosa but decreased iron, zinc, manganese, copper, and cadmium in the colonic lumen. Additionally, SLC39A8 A393T mice exhibited changes in microbiome composition and function associated with metal dishomoeostasis (130). SLC39A8 A393T mice have also been shown to have increased susceptibility to colitis (131, 132).

Specific Zn transporters have been identified as having key functions in maintaining gut barrier health. In mice lacking functional ZIP4 in intestinal epithelial cells, Paneth cells became Zn deficient, displayed reduced Sox9 and lysozyme expression, and resulted in disorganization of villus and crypt structures (133). ZIP7 has been found to be essential for the maintenance of the intestinal stem cell niche. Mice lacking ZIP7 in intestinal epithelial cells exhibited increased endoplasmic reticulum (ER) stress in progenitor cells, resulting in cell death. Additionally, there was a decrease in Olfm4^+^ stem cells and a degeneration of Paneth cells (134). ZnT2, which is localized on Paneth cell granules, has also been identified as important to Paneth cell function. The deletion of ZnT2 results in degranulation, less active lysozyme, reduced bactericidal activity, and alterations in gut microbial composition (135). Interestingly, deletion of ZnT2 resulted in improved disease outcomes in a model of infectious colitis by colonization with *Citrobacter rodentium*. Mice lacking ZnT2 exhibited less colonic inflammation, did not experience colon hyperplasia, and retained their goblet cells. These improvements in disease outcome may be at least partially mediated by reduced expression of TLR4. Studies in a human colonocyte cell model, HT29 cells, showed that knockdown of ZnT2 resulted in less TLR4 expression and, in turn, less nuclear translocation of NF-κB (136). Deletion of ZnT7, which is localized on the membrane of the Golgi complex, resulted in sex-dependent differences in mucin production, goblet cell counts, and microbiome composition. Male mice lacking ZnT7 exhibited increased goblet cell counts and higher mucin density compared to wild-type (WT) mice. In contrast, female mice lacking ZnT7 exhibited decreased goblet cell counts and mucin density compared to wild-type (WT) mice (137). ZIP14 is localized to the basolateral membrane of intestinal epithelial cells and has been found to regulate barrier integrity. Of note, ZIP14 is most closely related to ZIP8, which has been implicated in CD (58, 129, 138). The deletion of ZIP14 resulted in a reduction in the expression of tight junction proteins occludin and claudin-1, accompanied by an increase in the expression of claudin-2, leading to decreased gut permeability (61, 139). In mice lacking ZIP14, specifically in intestinal epithelial cells, the expression of proinflammatory genes, including IL-6 and IFN-*γ*, was increased. Histone deacetylase function was impaired, and a decrease in major histocompatibility complex class II was observed, suggesting that ZIP14-mediated Zn transport may impact gut barrier function by epigenetic mechanisms (139, 140). Loss of ZIP14 does not result in spontaneous gastrointestinal disease; however, it has been implicated in potential precursors to chronic disease, including gut permeability, alterations in host metabolism, and shifts in the gut microbiome similar to those observed in IBD. Specifically, the global deletion of Zip14 in mice led to an increased ratio of fungi to bacteria in the fecal microbiome, at least partially driven by an increase in *Saccharomyces cerevisiae,* which has been linked to IBD (141, 142). A decrease in *Akkermansia muciniphila* was also observed in mice lacking ZIP14, in line with observations in patients with IBD, as well as those with obesity and diabetes (141). ZIP14 has also been implicated in dietary risk factors for IBD. In mice administered a subchronic sucrose treatment through drinking water, ZIP14-mediated changes in Zn homeostasis were associated with an imbalance in epithelial turnover marked by increased epithelial proliferation, decreased apoptosis, and increased gut permeability (15). Collectively, Zn transport proteins have recently been identified as playing a role in maintaining intestinal health, although few have been studied specifically in the context of IBD.

## 2 Discussion

**Figure 2:**
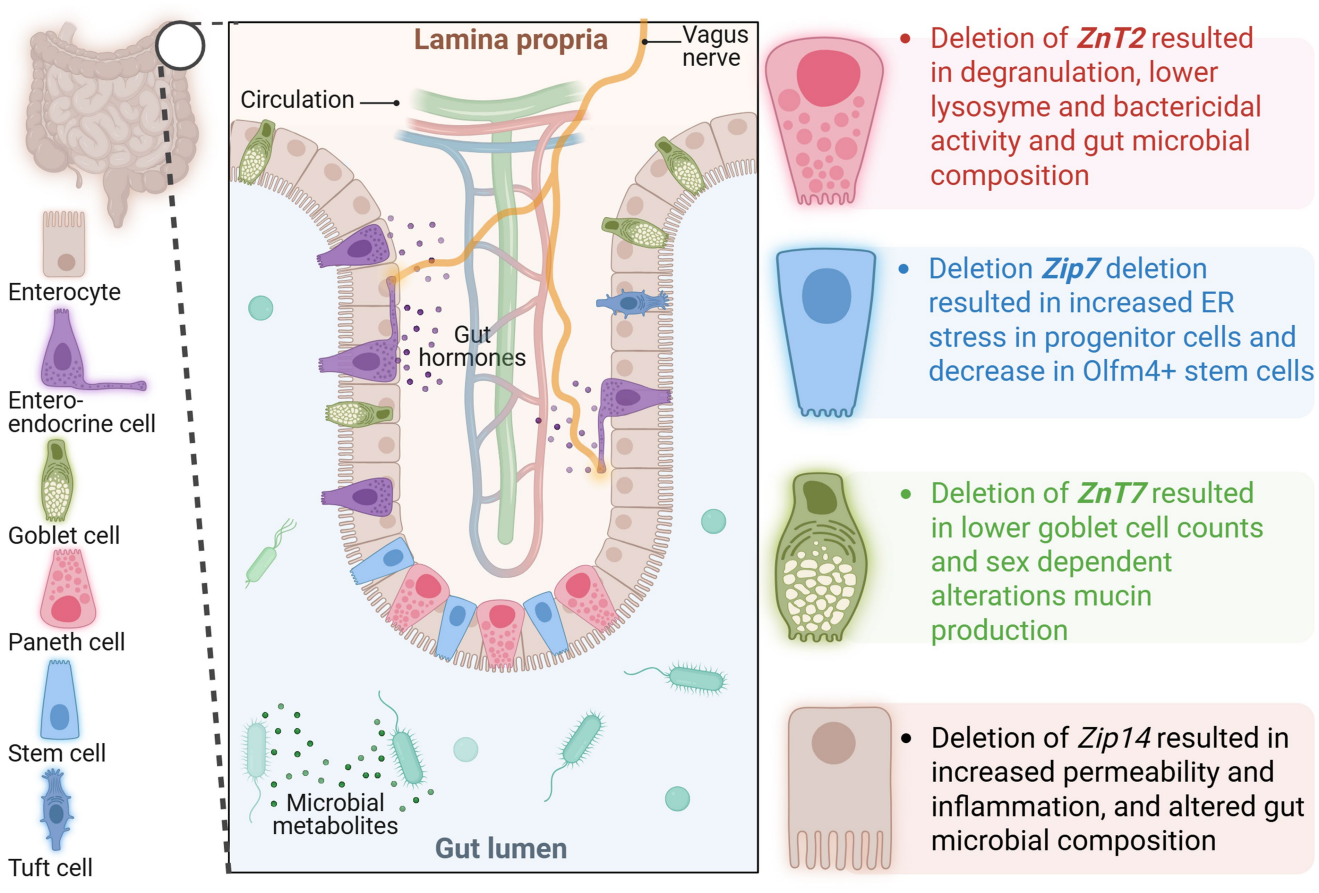
Depiction of an intestinal villus/crypt with color-coded specialized epithelial cells and known function of Zn transporters in each cell type based on the data from gene deletion studies. ZIP, Zrt-Irt-Like Proteins; ZNT, Zinc Transporter.

Zn deficiency is common in IBD patients and is associated with worse clinical outcomes, including increased hospitalizations, higher risk of complications, and disease severity. The precise role of Zn in IBD pathogenesis, however, remains unclear; whether deficiency is a cause or consequence of the disease is still an open question. Recent studies have highlighted the key roles of Zn transporters, such as ZIP4, ZIP7, ZIP8, ZIP14, ZnT2, and ZnT7, in maintaining intestinal health by regulating processes including tight junction expression, Paneth cell function, mucin production, gut permeability, and gut microbial diversity and composition (Figure 2). These findings suggest Zn transport dysregulation, driven by genetic, inflammatory, or dietary factors, may be a critical contributor to the impaired gut barrier and immune dysregulation characteristic of IBD. While Zn supplementation has shown potential to improve outcomes in some cases (Table 1), variability in results highlights the need for further mechanistic understanding of the reciprocal mechanistic regulation between Zn metabolism and IBD.

*Lack of a Reliable Biomarker for Zn Status*: Assessing Zn deficiency remains difficult due to the absence of specific and sensitive biomarkers. Serum Zn levels, the most commonly used measure, are influenced by inflammation, recent dietary intake, and other confounding factors, rendering them unreliable indicators, especially in chronic diseases such as IBD. Beyond circulating Zn levels, gaining a mechanistic understanding of inflammation-induced tissue-, cell-, and organelle-specific alterations in Zn metabolism and the whole metallome profile in a disease at all stages is necessary to link the function of Zn to specific cellular events, thus identifying better candidates for inflammation-dependent and independent biomarkers. Achieving this goal requires an integrated approach combining basic and translational science. This includes *in vivo* studies using diverse preclinical disease models alongside ex vivo organoid and cell culture systems derived from both animal models and human subjects.

*Understanding Zn Transporter Mechanisms*: While Zn transporters have been implicated in IBD and other diseases, much remains unknown about their precise functions, regulatory mechanisms, and tissue/cell-specific roles. For example, the missense variant in SLC39A8, which results in the dysregulation of multiple elements in various organ systems, has been shown to alter the gut microbial composition and confer a risk for Crohn’s disease, highlighting the essentiality of functional transport activity in gastrointestinal tissues. However, besides ZIP8, studies investigating the mechanistic link between Zn transporters in IBD are scarce. As mentioned in the earlier sections, the regulation of these transporters is multifaceted, responding not only to Zn levels but also to various cytokines, hormones, secondary messengers, and dietary components (8, 13–16, 62, 143–149). These modes of regulation suggest that disease-induced dysregulation in Zn transporters may contribute to worsening disease outcomes. Advancements in RNA sequencing have opened new avenues for analyzing cell-type-specific expression patterns of Zn transporters in human samples from both healthy individuals and patients with IBD. This technology holds immense promise for uncovering previously unrecognized links between Zn transporter dysfunction and IBD. Future research should prioritize the identification of Zn transporters linked to gastrointestinal health. Mechanistic studies utilizing genetically modified preclinical animal models will be particularly crucial, as they enable in-depth investigations into the roles of Zn transporters in distinct regions of the gastrointestinal tract and specific cell types within those regions while facilitating metallomics analyses to establish accurate reference values for Zn distribution across tissues, cells, and organelles. Additionally, the emerging technique of spatial transcriptomics and imaging, which integrates advanced imaging technologies with omics analyses, offers an innovative approach to profile Zn transporter expression spatially. These approaches could be used to detect changes in intestinal cell type abundance, cellular pathways, gene regulatory networks, and cell–cell signaling, enabling the identification of Zn transporters that modulate pivotal pathways and gene networks implicated in IBD. Such multidisciplinary efforts could provide invaluable insights into the causal roles of Zn transporters in IBD pathophysiology and the development of effective therapeutic interventions.

*Role of the Microbiome in Zn Uptake and IBD*: The relationship between Zn status, microbiome composition, and IBD remains incompletely understood. Zn has been shown to influence microbial diversity and functionality, but further research is needed to clarify how Zn-driven changes in the microbiome might promote or alleviate intestinal inflammation. Multi-omics approaches integrating metagenomics, transcriptomics, metabolomics, and metallomics may provide crucial insights.

Addressing these gaps will require multidisciplinary approaches that integrate nutrition science, biochemistry, systems biology, and clinical research. A deeper understanding of Zn metabolism and its interactions with host genetics, immune functions, and the microbiome will pave the way for more effective Zn-based therapeutic and preventive strategies for IBD and other chronic inflammatory diseases. Continued efforts in these areas will contribute to a broader understanding of the essential role of Zn in optimizing human health within the context of modern nutritional and medical challenges.
